# Doctors Perceptions Regarding Electronic Systems at Primary Healthcare Centers in Qassim Region, Saudi Arabia

**DOI:** 10.7759/cureus.48536

**Published:** 2023-11-08

**Authors:** Emad F Aljarbooa, Chandra Sekhar

**Affiliations:** 1 Family and Community Medicine, Qassim Health Cluster, Buraydah, SAU; 2 Family and Community Medicine, Family Medicine Academy, Qassim Health Cluster, Buraydah, SAU

**Keywords:** saudi arabia, communication, time efficiency, electronic health systems, primary health care centres, physicians

## Abstract

Background: Close to three-fourths of the population's health problems can be managed by way of providing care and prevention strategies at the primary healthcare center (PHCC) level. Periodical collections of physicians' perceptions about electronic systems at primary healthcare centers are required for quality improvement and also for the benefit of community practice. The objectives of the study are to find the physicians' perceptions, performance, and communication status with electronic systems.

Methodology: A cross-sectional study was conducted among the PHCC physicians during the period from September 2022 to October 2023. Data was collected through semi-structured, self-administered electronic forms distributed to physicians based on the criteria. Data was entered, cleaned, and analyzed with Statistical Package for Social Sciences (SPSS) version 20 software (IBM Corp., Armonk, New York). Necessary statistical tests like proportions and chi-square tests were applied.

Results: In the present study, 135 primary healthcare physicians participated. The response rate in the study population was 95%, and the mean age and the standard deviation in the study population were 32.99 ± 8.05. Approximately 91.1% of the physicians expressed that systems are essential in their daily work. Also, a similar percentage (90%) was observed about improving the quality of care. In contrast to this result, 60.8% of the physicians mentioned increased productivity, and decision-making was about 61.5%.

Conclusions: Based on the study findings, physicians gave good perceptions about electronic systems working at PHCC. However, certain domains need to be improved in regard to decision-making and increasing productivity.

## Introduction

In today's competitive global landscape, the future growth and development of a nation hinge on technological advancements, which can reduce time and human resources. Regular enhancements in systems and workforce capabilities are paramount. From this standpoint, electronic health record (EHR) systems serve as repositories for comprehensive patient data. This includes laboratory and radiological investigations, outpatient and inpatient clinical notes, recommendations, and medication histories. The EHR system stands out as an invaluable tool, aiding healthcare professionals in decision-making and ensuring that they deliver coordinated and optimal health services to their patients.

Electronic health systems (EHS) have been part of our healthcare landscape for several decades. Specifically, within the primary healthcare tier, EHS was introduced in Qassim province only a few years ago, to the best of our knowledge. The system continues to evolve with each passing day. Numerous studies have been undertaken to evaluate its efficacy and its contribution to health systems both globally and nationally. A study by Holroyd-Leduc et al. found that electronic medical records (EMRs) and electronic health records (EHRs) have a mixed impact on primary care outpatient practices. While the EHR system offers structural and process advantages, its impact on clinical outcomes remains uncertain. When adopting an EHR system, it is imperative to allocate appropriate resources [1].

Another research carried out in the United States concluded that although health information technology (HIT) and EHR hold the potential to boost quality, EHR usage did not consistently correlate with enhanced quality in ambulatory care. When deciding on an EHR system, medical practices should meticulously weigh the inclusion of clinical decision-support features and tools that can foster quality care across patient populations. Advocates in the health sector routinely assess the caliber of medical care in tandem with the expanding use of EHR in healthcare delivery [2].

In the study by Koopman et al., the design of the EHR was discussed, revealing that the current ambulatory progress notes present more information to the physician than is necessary [3]. It is essential to reconstruct the clinic progress note to match the workflow and information needs of its primary consumers. A 2017 study emphasized the importance of regular evaluation and monitoring of EHR. It also highlighted the need for significant developmental work to achieve the anticipated benefits of EHR systems, prevent technology-induced errors, and maintain patient safety measures [4]. The clinicians' perspectives as EHR end-users are crucial, as the success of the implementation often hinges on how this change manifests in the daily routines of physicians and nurses working with the EHR systems.

Another study explored the perceptions of health workers post-EHR implementation, concluding that the findings indicate satisfaction with an ambulatory EMR system implemented in a pediatric emergency department and urgent care clinics. The results underscored the importance of technical support. Consequently, system implementation strategies should focus on ensuring positive initial impressions of training, support, and performance to maximize the chances of achieving high user satisfaction [5].

Kaipio et al.'s 2020 study examined physicians' and nurses' experiences with EHR usability. They found that the overall usability ratings were low, aligning with previous research. While nurses expressed concerns about the learnability and stability of the systems, physicians were critical of the system's ease of use and ability to facilitate collaboration [6].

A study from the United Arab Emirates (UAE) by Al Alawi et al. concluded that clinicians in primary healthcare centers (PHCCs) have a generally positive perception of the EMR application in outpatient practices [7]. This research identified key areas to enhance the EMR system's benefits to patients, such as adapting the system to the needs of the primary healthcare setting and providing ongoing training and technical support, which could help reassure hesitant EMR users.

Research from the Makkah region revealed the mixed impacts of EMRs. The findings suggested that physicians are generally satisfied with EMRs and believe it can enhance the quality of care [8]. For the comprehensive development of the EMR, the collaboration of all stakeholders, including healthcare professionals and IT support, is essential. Future strategies should focus on system improvements, considering continuous upgrades, cost factors, and areas identified for enhancement by the Ministry of Health.

A separate study in Saudi Arabia indicated that physicians were largely dissatisfied with the EMR system, emphasizing that multiple aspects of the system need refinement [9]. Periodic evaluations of the installed systems and consistent feedback can guide the future selection and rollout of EMR systems. Despite significant investments in health information systems and the Ministry of Health's push for widespread EMR implementation, there is still room for improvement in Saudi hospitals.

In conclusion, while numerous researchers have explored EHR systems, there remains no consensus on its reception among health workers. Given these varying findings, our study aims to examine doctors' perceptions of EHR in PHCCs in the Qassim Region.

## Materials and methods

Study setting and target population

The study was conducted at PHCCs in Qassim, spanning three cities and one rural village. These locations account for nearly 80% of Qassim province's population. The target population consisted of doctors practicing in PHCCs throughout Qassim province and family medicine residents affiliated with Qassim Family Medicine Academy (FMA).

Study design

A cross-sectional approach was utilized, with data collection achieved via a self-administered electronic survey using a Google Form.

Data collection tool

For tool development, we reviewed studies available on search engines, finding a total of nine pertinent studies. Two of these studies mirrored our research idea [7,8]. After obtaining permission via email from the principal investigators of both studies, we incorporated some of their questionnaire variables into our study. Following this, we engaged in discussions with my supervisor and colleagues to enhance the questions, culminating in the creation of our questionnaire. The final product is a pretested, semi-structured questionnaire. This questionnaire consists of three primary sections: demographic data, doctors' perceptions of EHS, and domains pertaining to performance and communication.

Sampling

According to the Ministry of Health, there are 155 PHCCs operating within Qassim province, accommodating an estimated 568 primary care doctors, including 60 family medicine residents. Our study incorporated 25% of these physicians. Thus, our sample size was 142 doctors and residents. We selected PHCCs from three major cities-Buraydah, Unaizah, Alrass-and one rural region, Ueon Aljoua. The PHCCs from these areas were chosen using a simple random method. Typically, each center has approximately five physicians. As such, we selected 30 PHCCs to achieve our target of surveying 142 physicians. For random PHCC selection, we utilized the sample size calculator from https://www.calculatorsoup.com. Based on a list detailing physician availability, Google Forms were dispatched to the relevant primary healthcare doctors in Qassim province. The principal investigator was physically present until each questionnaire was completed. Prior to disseminating the questionnaire, informed consent was obtained, and the participants were assured of data confidentiality. All physicians in each PHCC who met our study criteria and expressed interest were considered.

Inclusion criteria

Primary healthcare physicians from selected cities within Qassim province of either gender.

Exclusion criteria

Physicians uninterested in participating, as well as those practicing in other Qassim cities or other Qassim hospitals, were excluded.

Pilot study

A pilot study was undertaken in the field to assess technical feasibility and to ensure proper question presentation and sequencing among 10 physicians. The pilot study's participants were not included in the primary study sample.

Ethical considerations

Before initiating data collection, we secured approval from the regional ethics committee (REC) in Qassim. Every stage of the research upheld strict ethical standards. Our research proposal, bearing approval number 607-44-6793 and dated November 24, 2022, was granted by the REC prior to the study's commencement. No participants were harmed during the study. Before beginning the research at selected PHCCs, permission was secured from the PHC director.

Statistical analysis

For continuous variables, means and standard deviations were computed. The chi-square test was applied to the categorical variables. A probability (P) value of ≤0.05 was designated as the threshold for statistical significance.

## Results

In our study, 135 primary healthcare physicians participated, yielding a response rate of 95% (135/142). For validity, all questions related to the perceptions, performance, and communication domains regarding electronic systems were analyzed using Statistical Package for Social Sciences (SPSS) version 20 software (IBM Corp., Armonk, New York). Cronbach's alpha was tested, yielding a value of 0.901. The mean age of the study population was 32.99, with a standard deviation of ±8.05. A notable majority of the physicians, 74.8%, were in the age group of 25-35 years, while a minority, 2.2%, were older than 55 years. Concerning qualifications, equal proportions were observed for MBBS and Diploma/board, each constituting 41.5%. In the study population, 58.5% of participants were residents or general physicians. The mean years of experience among the physicians was 6.13, with a standard deviation of ±6.70. Similarly, physicians, on average, had worked with 3.03 systems, with a standard deviation of ±1.23 (Table 1).

**Table 1:** Demographic characteristics of physicians at Qassim's PHCCs. PHCC: primary healthcare center.

Nationality	Number of participants	Percentage
Saudi	105	(77.8)
Non-Saudi	30	(22.2)
Age ± SD	32.99 ± 8.05	
Age category		
25–35 years	101	(74.8)
36–45 years	21	(15.6)
46–55 years	10	(7.4)
More than 55	3	(2.2)
Qualification		
MBBS	56	(41.5)
Diploma/board	56	(41.5)
Under-postgraduate program	23	(17.0)
Position		
Consultant	13	(9.6)
Specialist	43	(31.6)
Resident/GP	79	(58.5)
Years of experience ± SD	6.13 ± 6.70	
How many systems have you worked so far (number) ± SD 3.03 ± 1.23		

Table 2 evaluates the perceptions of physicians regarding electronic systems. The data indicates that the majority of physicians view these systems as essential in their daily medical care practice, with an observed mean ± SD of 4.53 (±0.75). For the statement "the system is helpful in my job performance," the mean perception and SD reported by physicians were 4.13 (±0.98). Concerning the statement "the system's style and information availability are clear to me," the lowest recorded mean and SD by physicians was 3.60 (±1.00).

**Table 2:** Perceptions of electronic systems among physicians in the study population.

Perceptions of physicians	Strongly disagree	Disagree	Neutral	Agree	Strongly agree	Mean SD	±
I consider electronic systems essential for daily medical care.	1 (0.7)	2 (1.5)	9 (6.7)	36 (26.7)	87 (64.4)	4.53 (±0.75)	
Electronic systems are helpful in improving the quality of care.	1 (0.7)	4 (3.0)	9 (6.7)	40 (29.6)	81 (60.0)	4.45 (±0.80)	
Electronic systems aid in decision-making.	3 (2.2)	15 (11.1)	34 (25.2)	43 (31.9)	40 (29.6)	3.76 (±1.06)	
The electronic system enhances my job performance.	3 (2.2)	6 (4.4)	21 (15.6)	45 (33.3)	60 (44.4)	4.13 (±0.98)	
The electronic system makes my job easier.	10 (7.4)	18 (13.3)	22 (16.3)	32 (23.7)	53 (39.3)	3.74 (±1.30)	
The style and information availability of the electronic systems are clear to me.	4 (3.0)	14 (10.4)	40 (29.6)	51 (37.8)	26 (19.3)	3.60 (±1.00)	
The benefits of the electronic systems outweigh the inconveniences.	3 (2.2)	7 (5.2)	33 (24.4)	52 (38.5)	40 (29.6)	3.88 (±0.97)	
Clear documentation within the electronic systems reduces errors.	1 (0.7)	2 (1.5)	13 (9.6)	57 (42.2)	62 (45.9)	4.31 (±0.76)	

Table 3 evaluates the domains of performance and communication among physicians. For the inquiry regarding whether previous training on systems would make their job easier, the mean and SD for physicians were 4.42 (±0.77). The lowest recorded mean and SD pertained to the question of whether systems assist in accomplishing tasks more swiftly, with a value of 3.20 (±1.30).

**Table 3:** Performance and communication domain status in a study group of physicians.

Performance and communication variables	Strongly disagree	Disagree	Neutral	Agree	Strongly agree	Mean ± SD
The systems assist in accomplishing tasks more quickly, such as no lags and speedy note and lab openings.	14 (10.4)	32 (23.7)	31 (23.0)	29 (21.5)	29 (21.5)	3.20 (±1.30)
The systems are deemed more time-efficient than traditional paperwork.	13 (9.6)	29 (21.5)	18 (13.3)	32 (23.7)	43 (31.9)	3.47 (±1.38)
The systems enhance productivity.	7 (5.2)	17 (12.6)	29 (21.5)	43 (31.9)	39 (28.9)	3.67 (±1.17)
Prior training on the systems would facilitate easier job performance.	1 (0.7)	2 (1.5)	12 (8.9)	44 (32.6)	76 (56.3)	4.42 (±0.77)
The systems are perceived as user-friendly.	2 (1.5)	17 (12.6)	42 (31.1)	52 (38.5)	22 (16.3)	3.56 (±0.95)
The systems streamline the prescription of medications.	5 (3.7)	17 (12.6)	17 (12.6)	35 (25.9)	61 (45.2)	3.96 (±1.19)
The systems can sometimes disrupt communication between healthcare professionals and patients.	2 (1.5)	20 (14.8)	30 (22.2)	45 (33.3)	38 (28.1)	3.72 (±1.07)
The systems modify communication methods with support departments like labs, radiology, and pharmacy.	6 (4.4)	12 (8.9)	28 (20.7)	58 (43.0)	31 (23.0)	3.71 (±1.05)

Table 4 presents the association of sociodemographic data with job performance and system clarity variables. In this analysis, observations originally recorded on a five-point Likert scale (strongly disagree, disagree, neutral, agree, and strongly agree) were converted to a three-point Likert scale (combining strongly disagree with disagree, keeping neutral unchanged, and combining agree with strongly agree).

Physicians in the 25-35 years age category responded with "agree" or "strongly agree" to the statement that the "system is helpful in my job performance" at a rate of 74.3%. For those in the 36-45 years age bracket, the response rate was 81.0%. Furthermore, both the 46-55 years age group and those aged more than 55 years unanimously responded with "agree" and "strongly agree" at a rate of 100%. No statistically significant association was observed between age category and the perception that the system is helpful in job performance (P=0.500).

Regarding the variable, "system style and information availability are clear to me," physicians aged 25-35 years responded with "agree" and "strongly agree" 48.5% of the time. The response rate for the 36-45 years age group was 76.2%. The 46-55 years age group and those older than 55 years had response rates of 90.0% and 100%, respectively. A statistically significant association was observed between higher age categories and the perception that system style and information availability are clear (P=0.040) (Table 4).

**Table 4:** Associations between demographic factors (age and position) and variables related to job performance and system clarity.

Demographic factors	The system is helpful in my job performance			
Age	Strongly disagree + disagree	Neutral	Agree + strongly agree	Total
25–35 years	7 (6.9)	19 (18.8)	75 (74.3)	101
36–45 years	2 (9.5)	2 (9.5)	17 (81.0)	21
46–55 years	0 (0.0)	0 (0.0)	10 (100)	10
>55 years	0 (0.0)	0 (0.0)	3 (100)	3
Total	9 (6.7)	21 (15.6)	105 (77.8)	135
X2—5.35, 6df, P-0.500				
The system is helpful in my job performance				
Position of doctor	Strongly disagree + disagree	Neutral	Agree + strongly agree	Total
Resident/GP	6 (7.6)	15 (19.0)	58 (73.4)	79 (100)
Specialist	3 (7.0)	6 (14.0)	34 (79.1)	43 (100)
Consultant	0 (0.0)	0 (0.0)	13 (100)	13 (100)
Total	9 (6.7)	21 (15.6)	105 (77.8)	135 (100)
X2—4.69, 4df, P-0.320				
	System style and information availability are clear to me			
Age	Strongly disagree + disagree	Neutral	Agree + strongly agree	Total
25–35 years	17 (16.8)	35 (34.7)	49 (48.5)	101 (100)
36–45 years	1 (4.8)	4 (19.0)	16 (76.2)	21 (100)
46–55 years	0 (0.0)	1 (10.0)	9 (90.0)	10 (100)
>55 years	0 (0.0)	0 (0.0)	3 (100)	3 (100)
Total	18 (13.3)	40 (29.6)	77 (57.0)	135 (100)
X2—13.17, 6df, P-0.040				
	System style and information availability are clear to me			
Position of doctor	Strongly disagree + disagree	Neutral	Agree + strongly agree	Total
Resident/GP	12 (15.2)	27 (34.2)	40 (50.6)	79 (100)
Specialist	6 (14.0)	12 (27.9)	25 (58.1)	43 (100)
Consultant	0 (0.0)	1 (7.7)	12 (92.3)	13 (100)
Total	18 (13.3)	40 (29.6)	77 (57.0)	135 (100)
X2 —8.06, 4df, P-0.089				

## Discussion

The integration of EHS into healthcare delivery, including PHCCs, is paramount. Such systems can significantly reduce human error, improve accountability, streamline the tracking of patient files, and provide various other facilities. With the inevitable global advancements in technology, the focus should be on leveraging it appropriately to reduce manpower yet enhance efficiency. This cross-sectional study assessed the perceptions of physicians working at PHCCs regarding the usability, performance, and communication domains of electronic systems at PHCCs from September 2022 to October 2023.

In this study, 74.8% of the study population were under 35 years of age. In contrast, a study encompassing the entire Qassim region found that 75% of physicians were under 45 years of age [10]. This minor age difference could be attributed to our study focusing on urban areas within Qassim province.

Remarkably, 91.1%, with a mean of 4.53 (±0.75) of the surveyed physicians acknowledged the indispensability of these systems in daily medical care. Another study in Makkah reported that 77.7% of physicians, with a mean response of 4.1 shared this sentiment [8]. This subtle variation in views concerning EHS may result from differing study environments, such as hospitals.

In our study, about 90%, 4.45 (±0.80) of PHCC physicians believed that these systems significantly enhance the quality of care. A US-based study conducted in 2009 after the introduction of EHS in PHCCs found that 63% of physicians initially believed in its potential to improve care quality. This figure rose to 86% a year later [11]. By now, we anticipate an even higher acknowledgment of its pivotal role in quality improvement. Similarly, a systematic review in Oman [12] cited cross-sectional studies by Asiri et al. [13] and Alasmary et al. [14], all of which agreed that EMR elevates the quality of patient care.

Concerning the role of EHR in aiding decision-making, approximately 61.5%, 3.76 (±1.06) of physicians in our study concurred. A study from India involving community physicians corroborated our findings [15]. Furthermore, when evaluating whether the benefits of such systems outweigh the occasional hassles, 68.1%, 3.88 (±0.97) of our surveyed physicians were in agreement. A similar question in the Makkah study found that 47% of physicians echoed this sentiment [8].

Lastly, regarding the system's potential to mitigate errors, 88.1%, 4.31 (±0.76) of the physicians agreed. This is in alignment with another study in which 84% of physicians believed computers could minimize medical blunders [11]. Reinforcing this belief, a separate study in Alabama, USA, observed that incorporating clinical decision support (CDS) into computerized provider order entry (CPOE) could curtail medication errors by up to 86% [16].

"Is the system helpful in accomplishing tasks more quickly?" In response to this question, about one-third 34.1%, 3.20 (± 1.30) of the physicians disagreed. A study conducted in the UAE found that EMRs were time-consuming, which aligns with our findings [7]. In response to the question, "Does it increase my productivity?" about 60.8%, 3.67 (±1.17) of physicians agreed. In contrast, an Iranian study highlighted obstacles related to EHS, one of which suggested that EHR implementation could reduce productivity and disrupt workflow [17].

"Would prior training on systems make my job easier?" Approximately 88.9%, 4.42 (±0.77) of the doctors agreed with this sentiment. Yet, a systematic review from Sweden revealed clinicians' perceptions that training is seen as an additional burden and results in extra workload [18-21].

Regarding the system's impact on prescribing medications to patients, approximately 83.7%, 3.74 (±1.30) of physicians rated it average or above. Around 91.6% of physicians in Qassim, Saudi Arabia, expressed average or higher satisfaction with the Wasfaty service, a centralized electronic medication prescription system [10]. This slight discrepancy may stem from the physicians' evolving interactions with electronic systems as technology and practices continually evolve.

"Do the systems interrupt the communication flow between you and the patients?" In response, roughly 61.4%, 3.72 (±1.07) of the physicians agreed. A study from Canada postulated that such systems might disrupt communication and the flow of information between healthcare providers and patients [22]. On the topic of whether systems altered communication with supportive departments (such as lab, radiology, and pharmacy), nearly 66%, 3.71 (±1.05) of doctors agreed. An Ethiopian study disclosed that departments such as the laboratory and pharmacy cited poor system usage by other healthcare professionals [23].

Our study had some limitations. During data collection, many physicians were occupied with their regular duties and patients, making it challenging for the principal investigator and data collector to meet with them. This also resulted in extended waiting periods. Geographic challenges, such as the vast distance between cities, presented additional obstacles. As our questionnaire was self-administered online, some questions might have been unclear to the participants, leading to potential biases. However, the principal investigator was consistently available to clarify doubts and answer inquiries throughout the data collection phase.

## Conclusions

Based on our study findings, there was positive perception, performance, and communication regarding EHS in PHCCs. Our results aligned favorably with many studies conducted elsewhere. Moreover, certain aspects of our results mirrored findings from studies in other parts of the world. However, there remain areas for improvement, particularly concerning decision-making, productivity enhancement, and uninterrupted communication between physicians and patients.
